# Elucidating key targets and mechanisms of diethyl phthalate-induced colorectal cancer through network toxicology and molecular docking

**DOI:** 10.1371/journal.pone.0343038

**Published:** 2026-02-17

**Authors:** Zijing Wang, Liyuan Ma, Zhanyuan Sun, Hengyi Lv, Ruxue Ma, Mengqi Ding, Hai Li, Tao Jiang

**Affiliations:** 1 First Clinical Medical College, General Hospital of Ningxia Medical University, Yinchuan, China,; 2 Department of Ultrasonography, General Hospital of Ningxia Medical University, Yinchuan, China; 3 Department of Nursing, School of Medical Teaching, Gansu University of Chinese Medicine, Lanzhou, Gansu, China; 4 Department of Anal-Colorectal Surgery, General Hospital of Ningxia Medical University, Yinchuan, China; The University of Lahore - Raiwind Road Campus: The University of Lahore, PAKISTAN

## Abstract

**Background:**

Diethyl phthalate (DEP), a widely used plasticizer with endocrine-disrupting properties, has raised concerns regarding its potential carcinogenic effects. However, its precise role in colorectal cancer (CRC) development remains poorly understood.

**Methods:**

The chemical structure of DEP was obtained from the PubChem database. Potential targets of DEP were identified through ChEMBL and STITCH databases and intersected with known CRC-related genes to screen for candidate biomarkers. Gene Ontology (GO) and Kyoto Encyclopedia of Genes and Genomes (KEGG) enrichment analyses were performed to explore the biological functions and signaling pathways involved. Molecular docking was conducted to predict the binding affinities between DEP and core targets. Finally, 200-ns molecular dynamics (MD) simulations using GROMACS were employed to evaluate the binding stability and dynamic behavior of the DEP–target complexes.

**Results:**

A total of 62 overlapping genes were identified between DEP targets and CRC-associated genes. GO and KEGG enrichment analyses indicated enrichment in epigenetic regulation, chromatin remodeling, and cancer-related signaling pathways, including Notch, TGF-β, and FoxO. Protein–protein interaction analysis identified EP300, EZH2, HDAC1, HDAC2, and KDM1A as key epigenetic regulators. Molecular docking predicted moderate binding affinities between DEP and these targets (−6.6 to −5.7 kcal·mol ⁻ ¹). Subsequent 200-ns MD simulations suggested that DEP formed stable complexes with HDAC1, KDM1A, and EZH2, moderate stability with EP300, and partial dissociation with HDAC2, consistent with hydrophobic and hydrogen-bonding interactions at the binding interfaces.

**Conclusion:**

This study provides a theoretical framework for exploring the molecular mechanisms through which DEP may contribute to CRC development, emphasizing the value of network toxicology in cancer research. These findings may inform future investigations into the risks of DEP exposure and support public health policy and the development of targeted therapeutic strategies.

## 1. Introduction

Diethyl phthalate (DEP) is a widely used plasticizer primarily employed to enhance the flexibility and ductility of plastic products, and it is also frequently present in cosmetics and personal care items [1,2]. Owing to its chemical stability and low volatility, DEP persists in the environment and bioaccumulates in living organisms, raising growing concern among both the public and the scientific community [3,4]. As an environmental endocrine disruptor, DEP can mimic or interfere with endogenous hormonal signaling, thereby disturbing endocrine homeostasis. Such dysregulation may lead to reproductive abnormalities, metabolic disorders, and imbalances in cell proliferation and differentiation, which may ultimately contribute to increased cancer risk [5]. Beyond its endocrine-disrupting effects, accumulating toxicological evidence suggests that DEP and structurally related phthalates can induce oxidative stress, DNA damage, and inflammatory responses in intestinal and hepatic cells [6,7]. These toxic effects are frequently mediated through the activation of MAPK, NF-κB, and Wnt/β-catenin signaling pathways, which are well recognized for their pivotal roles in colorectal carcinogenesis [8,9]. Furthermore, DEP exposure has been shown to alter tight junction protein expression and compromise epithelial barrier integrity, potentially facilitating epithelial–mesenchymal transition (EMT) and malignant transformation [10,11]. Recent studies have also demonstrated that DEP can upregulate cyclin D1 expression via activation of the PI3K/AKT signaling pathway [12]. Cyclin D1, a key regulator of the G1-to-S phase transition, is closely associated with the pathogenesis of various malignancies. Collectively, these findings indicate that DEP may exert carcinogenic potential through multiple molecular pathways, affecting not only endocrine regulation but also critical processes governing tumor initiation and progression.

Colorectal cancer (CRC) remains one of the leading causes of cancer-related morbidity and mortality worldwide [13]. Its development involves a complex interplay of genetic susceptibility, lifestyle factors, and environmental exposures [14]. Among these, environmental pollutants such as polycyclic aromatic hydrocarbons (PAHs) are known to activate the aryl hydrocarbon receptor (AhR), driving epithelial proliferation and differentiation that favor tumorigenesis [15,16]. The lipophilic nature of PAHs enables their bioaccumulation, and long-term exposure has been linked to elevated CRC risk [17]. Given that DEP exhibits similar lipophilic properties and environmental persistence, and that other phthalates such as di(2-ethylhexyl) phthalate (DEHP) and its metabolite mono(2-ethylhexyl) phthalate (MEHP) have been reported to enhance proliferation, migration, EMT, and chemoresistance in colon cancer cells, DEP may contribute to CRC development.

Nevertheless, despite these mechanistic indications, no direct in vivo or epidemiological evidence currently links DEP exposure to CRC. This knowledge gap highlights the necessity of a systematic investigation into DEP’s potential role in colorectal carcinogenesis. Network toxicology offers a powerful framework to elucidate the complex interactions between environmental chemicals and biological systems through integrative molecular network analysis [18]. By combining bioinformatics, cheminformatics, and computational modeling, this approach enables prediction of chemical–target interactions and associated toxicological pathways [19]. In the present study, we employed network toxicology, molecular docking, and molecular dynamics simulations to identify potential key targets and signaling pathways underlying DEP-induced carcinogenicity. Our findings aim to provide mechanistic insights into how DEP may contribute to CRC development and to guide future experimental validation.

## 2. Method

### 2.1 Compound structure acquisition and toxicity analysis

The chemical structure and SMILES string of the target compound, DEP, were retrieved from the PubChem database (https://pubchem.ncbi.nlm.nih.gov/). Toxicity predictions were then performed using ProTox (https://tox.charite.de/), which employs a support vector machine (SVM)-based method to evaluate LD50 values, classify toxicity categories, and identify potential toxicological targets [20]. Additionally, comprehensive ADME (Absorption, Distribution, Metabolism, and Excretion) characteristics and toxicity analysis were conducted using ADMETlab (https://admetmesh.scbdd.com/), which covers assessments of hepatotoxicity, carcinogenicity, and reproductive toxicity [21].

### 2.2 Identification of compound target genes

Potential DEP target genes were retrieved from three databases: ChEMBL (https://www.ebi.ac.uk/chembl/), STITCH (http://stitch.embl.de/), and SwissTargetPrediction (http://www.swisstargetprediction.ch/). ChEMBL provides curated chemical–target interaction data, STITCH integrates known and predicted chemical–protein associations, and SwissTargetPrediction infers targets based on chemical structure similarity [22]. For STITCH, interactions with a confidence score >0.4 (medium confidence) were retained, consistent with thresholds commonly applied in prior studies [23]. For SwissTargetPrediction, targets with probability ≥0.1 were selected, following established practice to balance sensitivity and specificity [24]. The union of gene sets from the three databases was constructed, duplicates were removed, and Venn diagrams were generated using the R package *ggvenn* to illustrate target distribution.

### 2.3 Identification of CRC target genes

To identify CRC-associated target genes, we used GeneCards (https://www.genecards.org), OMIM (https://www.omim.org), and the Therapeutic Target Database (TTD, http://db.idrblab.net/ttd/). GeneCards provides comprehensive annotations for human genes, including gene function, expression, and disease associations. Genes were retrieved from GeneCards using the keyword “colorectal cancer” (restricted to *Homo sapiens*), and only those with a Relevance score ≥10 were retained, consistent with thresholds used in previous studies [25]. OMIM provides curated information on gene–disease associations [26], while TTD includes therapeutic target data such as drug–target interactions [27]. The union of gene sets from the three databases was constructed, duplicates were removed, and Venn diagrams were generated using the R package *ggvenn* to illustrate gene distribution.

### 2.4 Construction of compound regulatory networks

To identify potential biomarkers shared between DEP and CRC, we performed an intersection analysis and used the R package “ggvenn” to generate Venn diagrams displaying the overlap of genes between DEP and CRC. We then constructed a regulatory network of these genes using Cytoscape software [28], which visualized the interactions between these genes and proteins. Network analysis was performed to identify key biomolecules and biological pathways within the network.

### 2.5 Enrichment analysis

To explore the biological functions and pathways associated with the core gene set, Gene Ontology (GO) and Kyoto Encyclopedia of Genes and Genomes (KEGG) enrichment analyses were performed using the R package clusterProfiler [29,30]. Both the nominal p-value cutoff (*p* < 0.05) and the adjusted p-value cutoff (FDR < 0.05, Benjamini–Hochberg correction) were applied to identify significantly enriched terms. Enrichment categories included biological processes (BP), cellular components (CC), molecular functions (MF), and KEGG pathways [31]. Enrichment results were visualized using the ggplot2 package to highlight the functional characteristics of the core gene set.

### 2.6 Constructing the PPI network

The 62 overlapping genes between DEP- and CRC-related targets were imported into the STRING database to construct a protein–protein interaction (PPI) network. The analysis was restricted to *Homo sapiens*, with the minimum required interaction score set to >0.7 (high confidence). All available evidence channels in STRING were activated, including textmining, experimental data, curated databases, co-expression, neighborhood, gene fusion, and co-occurrence. No additional interactors were added. Interactions were defined by the combined score, which integrates both experimentally validated and predicted edges. The resulting network was exported from STRING and visualized in Cytoscape. The complete PPI edge list is provided in S1 Table. Network topology was analyzed in Cytoscape to evaluate the importance of nodes. Four centrality measures were computed: degree centrality, betweenness centrality, closeness centrality, and clustering coefficient. Among these, degree centrality was considered the primary determinant of node importance, as it directly reflects the hub characteristics of genes within the network [32]. Betweenness, closeness, and clustering coefficient were used as supportive parameters to confirm the robustness of the network structure. Based on these analyses, five molecules with high degree values and consistent centrality characteristics were identified as core nodes and selected for subsequent analysis. Detailed centrality values and rankings for all nodes are reported in S2 Table.

### 2.7 Molecular docking of DEP with core genes

To explore the potential interactions between DEP and CRC–associated target proteins, molecular docking analyses were conducted using the CB-Dock2 platform (http://cadd.labshare.cn/cb-dock2/). This platform integrates the *CurPocket* cavity-detection algorithm with the *AutoDock Vina 1.2.0* scoring engine, enabling automatic identification of potential binding pockets on protein surfaces and subsequent docking simulations [33]. The three-dimensional (*3D*) structures of the target proteins were obtained from the RCSB Protein Data Bank (PDB), and their corresponding PDB IDs, chain identifiers, and resolutions are summarized in S3 Table. Upon uploading the receptor, the platform automatically performed structural preprocessing, including the removal of water molecules, cofactors, and nonessential metal ions, followed by hydrogen addition and energy optimization to ensure conformational stability [34]. The *3D* structure of DEP (in mol2 format) was retrieved from the PubChem database and employed in docking simulations. The default parameters were set to *exhaustiveness* = 8 and *num_modes* = 20, and the binding affinity was evaluated using the *Vina* score (kcal·mol ⁻ ¹). For each target protein, multiple potential binding pockets were identified automatically, and the conformation with the lowest binding energy was selected as the optimal binding mode. The resulting optimal binding conformations were analyzed and visualized in three dimensions using PyMOL v2.6 to identify key binding interactions, including hydrogen bonds, hydrophobic interactions, π–π stacking, and π–alkyl interactions.

### 2.8 Molecular dynamics (MD) simulations

MD simulations were conducted using GROMACS 2022.2 [35]. The Amber14SB force field was applied to the protein [36], and the TIP3P model was used to describe water molecules [37]. The small molecule parameters were generated using Antechamber, with AM1-BCC charges and GAFF2 atom types assigned accordingly. The resulting topologies were converted into GROMACS format using ACPYPE [38]. Ion parameters were derived from the Joung–Cheatham set, which is compatible with the TIP3P water model. The protein–ligand complex was embedded in a truncated dodecahedral box, maintaining a minimum distance of 1.2 nm between the protein surface and the box boundaries. The system was solvated with TIP3P water molecules, and Na⁺ and Cl⁻ ions were added to neutralize the system and achieve an ionic strength of 0.15 M. Energy minimization was performed using the *Steepest Descent* algorithm until the maximum force (*F*_max_) dropped below 1000 kJ·mol ⁻ ¹·nm ⁻ ¹. Subsequently, the system underwent 200 ps of equilibration under both *NVT* and *NPT* ensembles to stabilize temperature and pressure. The production phase was performed for 200 ns in the *NPT* ensemble using a 2 fs integration time step. Long-range electrostatic interactions were computed using the *Particle Mesh Ewald (PME)* method [35], while both van der Waals and Coulombic interactions were truncated at 1.2 nm. All bonds involving hydrogen atoms were constrained with the *LINCS* algorithm. The temperature and pressure were maintained at *T* = 298 K and *P* = 1 bar using the *Nose–Hoover* thermostat and *Parrinello–Rahman* barostat, respectively. Simulation trajectories were saved every 10 ps. Trajectory analyses, including structural stability and intermolecular interaction assessment, were performed using GROMACS built-in tools, VMD, and PyMOL, while the binding free energy was calculated via the *gmx_MMPBSA* package when applicable.

### 2.9 Statistical analysis

All bioinformatics and statistical analyses were conducted using R software (version 4.3.2). A *p* value of < 0.05 was considered statistically significant. All database sources, software, and R package details (including versions, release dates, and URLs) are provided in S4 Table to ensure full transparency and reproducibility.

## 3. Result

### 3.1 Toxicity analysis of DEP

We conducted a comprehensive toxicity analysis of DEP using the ProTox and ADMETlab databases. The results indicate that DEP exhibits moderate toxicity to the kidneys and may interfere with drug metabolism by inhibiting multiple cytochrome P450 enzymes. Although DEP’s genotoxicity is low, its potential as an environmental endocrine disruptor and its impact on drug metabolic pathways suggest a possible mechanism through which DEP could indirectly influence the pathogenesis of CRC. These findings provide valuable insights into how environmental exposure can affect cancer progression through hormonal disruption and drug metabolism. Detailed toxicity prediction results from ADMETlab and ProTox are provided in S1 and S2 Files.

### 3.2 Identification of target genes common to CRC and DEP

**Fig 1:**
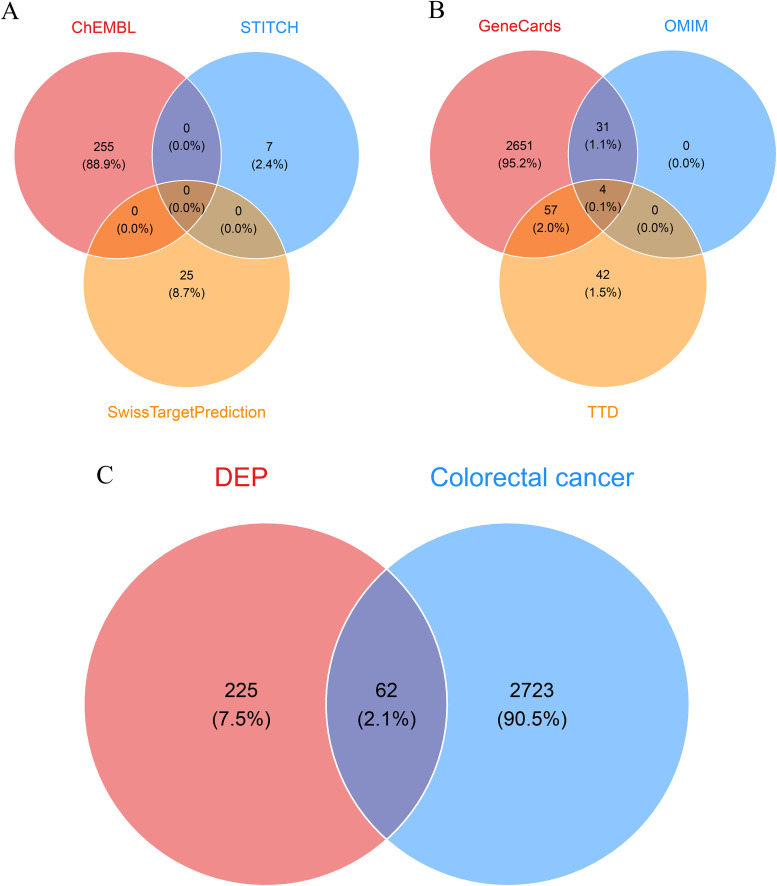
Screening potential targets of DEP in CRC. **(A)** The Venn diagram shows the cross-validation results of three different databases (ChEMBL, STITCH and SwissTargetPrediction) in DEP potential target screening. **(B)** The Venn diagram shows targets related to CRC in three databases: GeneCards, OMIM and TTD. **(C)** The Venn diagram directly linked DEP and potential targets for CRC, of which 62 targets (accounting for 2.1%) were associated with both DEP action and CRC, and these targets may be key mediators of DEP-induced CRC.

ChEMBL, STITCH, and SwissTargetPrediction yielded 255, 7, and 25 DEP-related target genes, respectively, with no overlap among the three sources (Fig 1A). The merged dataset comprised 287 unique DEP-associated genes (S5 Table). For CRC, 2,651 genes were obtained from GeneCards, 31 from OMIM, and 42 from TTD, with 31 shared between GeneCards and OMIM, 57 between GeneCards and TTD, and 4 common to all three databases (Fig 1B). After union and de-duplication, the CRC gene set consisted of 2,785 genes (S6 Table). Comparative analysis revealed 62 overlapping genes between DEP and CRC (Fig 1C), with 225 specific to DEP and 2,723 specific to CRC. The 62 overlapping genes are listed in S7 Table. These shared targets may contribute to DEP-induced CRC-related pathways, providing insights into potential carcinogenic mechanisms.

### 3.3 Regulatory network analysis of DEP and CRC shared target genes

**Fig 2:**
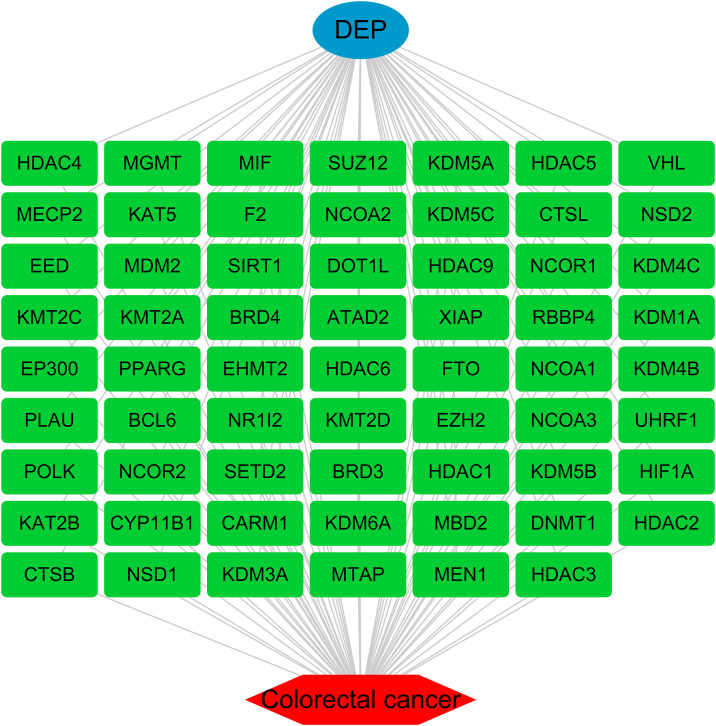
Network diagram of DEP and potential targets of CRC. Each green box in the figure represents a specific gene or protein. Starting from the central “DEP”, lines are connected to each target, indicating that these targets are associated with DEP action and may play a role in the pathological process of CRC.

By constructing a regulatory network, we identified potential shared target genes between DEP and CRC. This network revealed direct relationships between DEP and several key CRC-associated genes, including HDAC4, MGMT, PPARG, KMT2C, and BRD4, which play significant roles in tumorigenesis, cell cycle regulation, and epigenetic modifications (Fig 2). The diversity of these target genes suggests that DEP may influence CRC development and progression through multiple molecular pathways, underscoring DEP’s potential role in cancer-related signaling pathways.

### 3.4 GO and KEGG enrichment analysis results of DEP target genes in CRC

**Fig 3:**
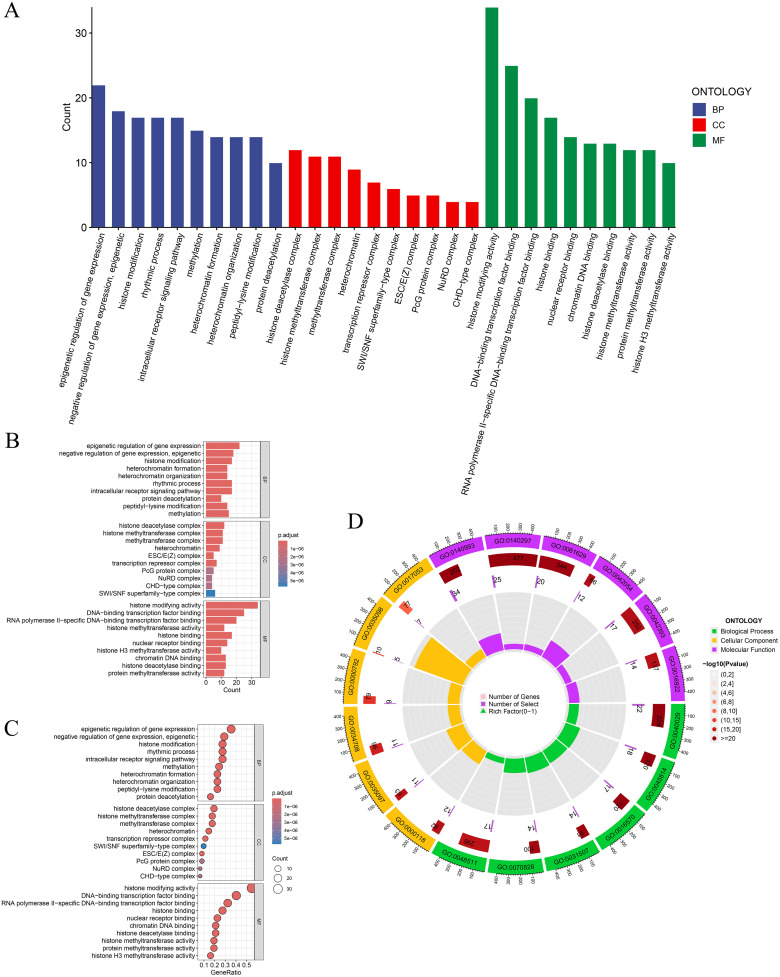
GO enrichment analysis results. **(A)** The bar chart shows the number of genes in the three categories of BP, CC, and MF in the GO analysis results, and different colors distinguish different categories. **(B-C)** Histograms and bubble plots show the significance of each functional category, and the shades of color indicate the significance level of the *p*-value. **(D)** The donut chart displays the significance and number of genes in the three main categories of GO in detail. From the inner circle to the outer circle, the number of genes, *p*-value and significance level are displayed in sequence.

This study identified enriched biological pathways and functions of potential DEP target genes in CRC through GO and KEGG enrichment analyses. In the BP category, DEP target genes were enriched in terms such as epigenetic regulation of gene expression, negative regulation of gene expression, and histone modification, indicating potential epigenetic involvement of DEP in CRC. In the CC category, enrichment was observed in histone deacetylase complex, methyltransferase complex, and transcription repressor complex, all associated with chromatin modification, suggesting that DEP may influence gene expression by modulating chromatin structure. In the MF category, DEP target genes were enriched in histone modification activity, transcription factor binding activity, and nuclear receptor binding activity, providing hypothesis-generating evidence that DEP might modulate transcriptional regulation in CRC (Fig 3).

**Fig 4:**
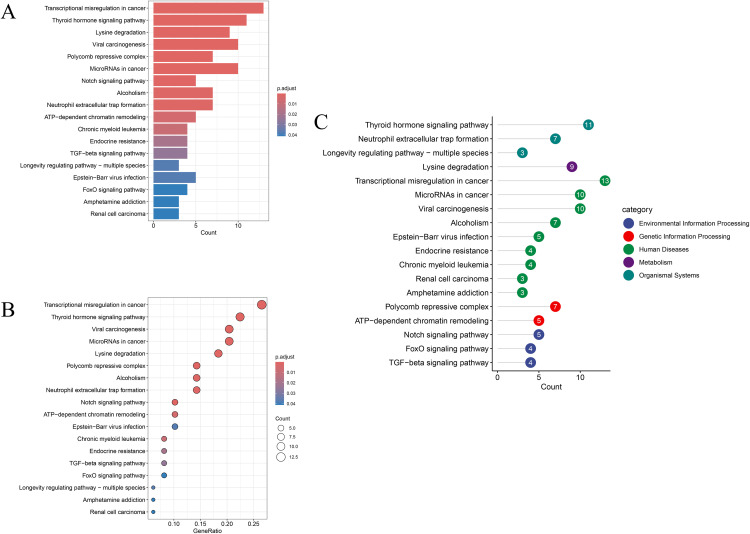
KEGG enrichment analysis results. **(A-B)** Bar charts and bubble charts show the degree of gene enrichment and adjusted p-value significance in different biological pathways, where the size of the points represents the number of genes in the pathway, and the depth of the color represents statistical significance. **(C)** This figure shows the results of different biological classification pathway enrichment based on the KEGG database. Each bar represents a specific biological pathway and is color-coded according to the biological category to which it belongs. The number next to each bar indicates the number of genes involved in the pathway.

KEGG enrichment analysis identified multiple enriched cancer-related signaling pathways, including transcriptional dysregulation in cancer, thyroid hormone signaling pathway, viral carcinogenesis, microRNAs in cancer, lysine degradation, and polycomb repressive complex. Enrichment in the Notch, TGF-β, and FoxO signaling pathways provided hypothesis-generating insights into potential mechanisms by which DEP exposure may affect cell proliferation, differentiation, apoptosis, and immune regulation (Fig 4). Detailed GO and KEGG enrichment results are summarized in S8 Table.

### 3.5 Role of key proteins in DEP-induced CRC

**Fig 5:**
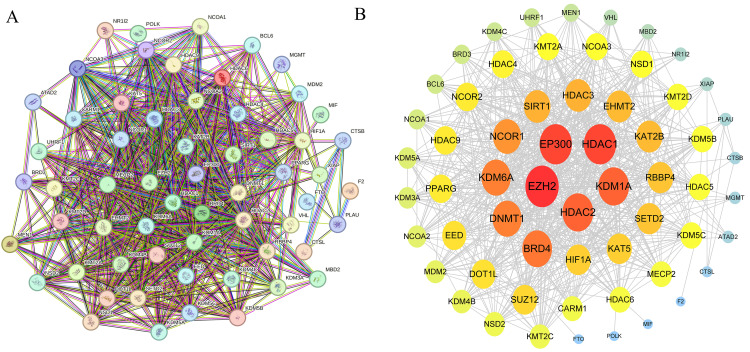
Protein interaction network and regulation center. **(A)** The PPI network diagram shows the complex interaction network between proteins, depicting how multiple different proteins are connected through direct interactions. Each colored node represents a unique protein, and the colorful lines indicate the type and strength of the interaction between proteins. **(B)** This figure shows the core components in the PPI network, with special emphasis on several key regulatory proteins such as EZH2, HDAC1, EP300, etc. Different colored nodes are used in the figure to distinguish the function or importance of proteins, among which the red nodes highlight the highly critical regulatory proteins that are the center of the network.

Using the PPI network constructed from the STRING database, we uncovered potential mechanisms by which DEP may induce CRC through specific protein interactions. Several hub-like nodes emerged from the network, including HDAC1, EP300, and EZH2, which are central regulators of gene expression, cell proliferation, and apoptosis (Fig 5A). Further analysis highlighted five core proteins—EP300, EZH2, HDAC1, HDAC2, and KDM1A—that exhibited high degree values and consistent centrality characteristics, marking them as key regulatory factors within the network (Fig 5B). Notably, EP300 and EZH2, both crucial in epigenetic regulation, stood out as particularly relevant to CRC. These findings suggest that DEP may contribute to CRC development by perturbing the functions of these core proteins and their interaction networks.

### 3.6 Molecular docking reveals interactions between DEP and epigenetic regulators

**Fig 6:**
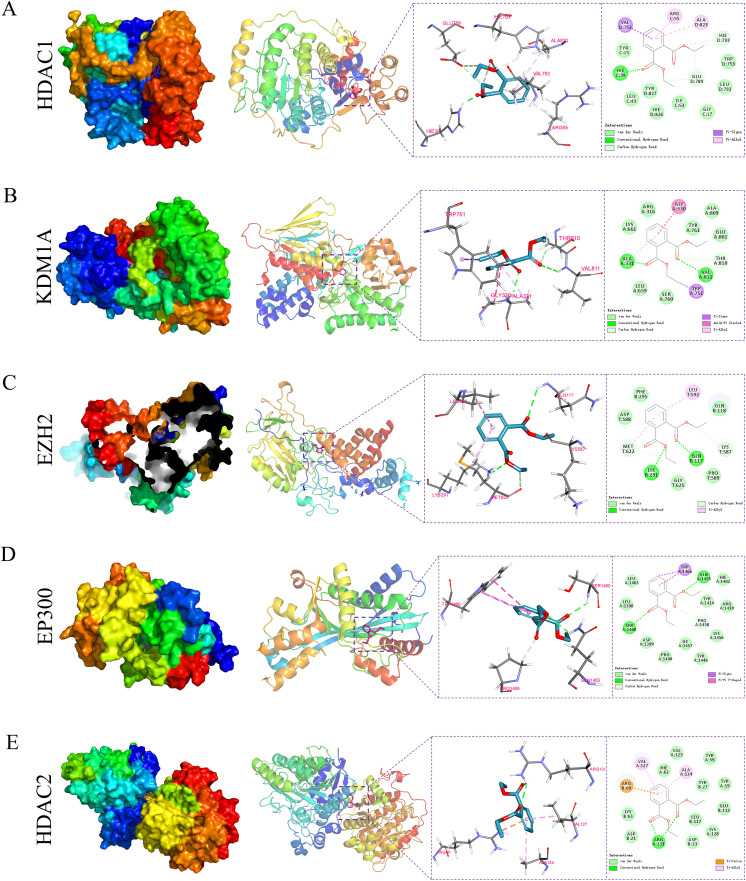
Molecular docking analysis of DEP with key epigenetic targets associated with CRC. The binding interactions of DEP with **(A)** HDAC1, **(B)** KDM1A, **(C)** EZH2, **(D)** EP300, and **(E)** HDAC2 are illustrated. Each panel displays the overall protein–ligand complex (left), the docking conformation within the active site (middle), and the detailed two-dimensional interaction map (right). DEP forms multiple hydrogen bonds, hydrophobic interactions, and π–π stacking with critical amino acid residues within the active sites of these enzymes.

To investigate the interactions between DEP and key CRC–associated target proteins, molecular docking analyses were performed on five core proteins: HDAC1, KDM1A, EP300, EZH2, and HDAC2. The calculated binding energies of DEP with these targets were −6.6, −6.6, −6.3, −5.7, and −5.8 kcal/mol, respectively, indicating that HDAC1 and KDM1A displayed the strongest binding affinities and thus the most stable interactions with DEP. Analysis of the binding interaction patterns revealed that DEP formed hydrogen bonds with key residues, including His39 in HDAC1, Ala331 and Val811 in KDM1A, Gln117 and Lys291 in EP300, Gln1455 and Ser1400 in EZH2, and Arg131 in HDAC2. In addition, DEP was stably accommodated within the active-site pockets of these proteins through multiple hydrophobic and aromatic interactions, such as π–sigma, π–alkyl, amide–π stacked, and π–π T-shaped interactions (Figs 6A–6E, S1 and S9 Table). Taken together, these findings suggest that DEP may interact with multiple epigenetic regulators involved in CRC, potentially influencing chromatin remodeling and gene transcription.

### 3.7 Stable interactions between DEP and epigenetic targets revealed by MD simulations

**Fig 7:**
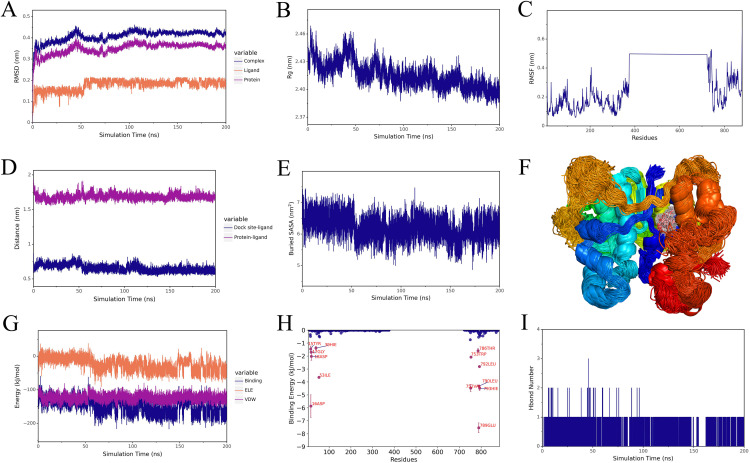
MD simulation analysis of the HDAC1–DEP complex. **(A)** RMSD of the complex, protein, and ligand; **(B)** Rg of the complex; **(C)** RMSF of protein residues; **(D)** distance between the binding site and ligand; **(E)** buried SASA; **(F)** superimposed conformations during simulation; **(G)** binding energy components (VDW and ELE); **(H)** per-residue binding energy contribution; and **(I)** variation in the number of hydrogen bonds during simulation.

To assess the binding stability between DEP and key epigenetic regulatory proteins, 200-ns MD simulations were performed for five protein–ligand complexes: EZH2, KDM1A, EP300, HDAC1, and HDAC2. The analyses included key parameters such as root mean square deviation (RMSD), radius of gyration (Rg), root mean square fluctuation (RMSF), ligand–protein distance, solvent-accessible surface area (SASA), hydrogen bonds, and MM-PBSA binding energy. All five complexes reached equilibrium during the simulations but exhibited distinct stability profiles. The HDAC1, EZH2, and KDM1A complexes were the most stable, with RMSD values converging to 0.20–0.30 nm after 30 ns and compact Rg values. RMSF analysis indicated limited fluctuations within the binding pocket, whereas larger fluctuations were mainly restricted to terminal or loop regions distant from the active site, suggesting that the local environments around DEP remained relatively rigid in these three complexes. The distance and SASA profiles changed smoothly, and the number of hydrogen bonds remained between one and two, reflecting stable ligand retention within the active site. The EP300 complex was stable during the early stages but exhibited slight fluctuations later, with RMSD increasing to approximately 0.35 nm and a binding energy of about −88.75 kJ·mol ⁻ ¹, indicating weaker binding affinity. The HDAC2 complex displayed the lowest stability, as shown by sharp increases in RMSD and SASA after 100 ns and the disappearance of hydrogen bonds, suggesting partial ligand dissociation. Its binding energy was only −40 kJ·mol ⁻ ¹. According to MM-PBSA calculations, the binding energy ranked as follows: HDAC1 (−105.49 kJ·mol ⁻ ¹)> KDM1A (−103.87 kJ·mol ⁻ ¹) ≈ EZH2 (−100.6 kJ·mol ⁻ ¹)> EP300 (−88.75 kJ·mol ⁻ ¹) ≫ HDAC2 (−40.92 kJ·mol ⁻ ¹). This trend indicates that DEP binding to HDAC1, KDM1A, and EZH2 is energetically more favorable, moderate to EP300, and clearly weakest to HDAC2. Van der Waals interactions were identified as the predominant driving force. Residue energy decomposition indicated that hydrophobic residues—mainly Leu, Val, Phe, and Tyr—were key contributors to complex stability (Figs 7A–7I and S2–S5). Among these complexes, the HDAC1–DEP complex combined the lowest RMSD, low RMSF in the binding pocket, and the most favorable MM-PBSA energy, indicating that DEP can be tightly accommodated in the hydrophobic pocket of HDAC1 and maintain persistent noncovalent interactions during the 200-ns simulation. In summary, DEP formed stable complexes with multiple epigenetic proteins, among which the HDAC1–DEP complex showed the strongest binding affinity, followed by EZH2 and KDM1A, while EP300 exhibited moderate stability and HDAC2 was unstable. These results suggest that DEP may selectively interact with specific epigenetic regulators implicated in CRC, with differences in binding stability and affinity that reflect its potential to differentially modulate epigenetic processes.

## 4. Discussion

DEP, a widely used plasticizer found in plastics, cosmetics, and personal care products, is classified as an environmental endocrine disruptor [39]. DEP can mimic or interfere with hormonal functions in the body, potentially impacting human health [40]. Long-term or high-dose exposure to DEP has been linked to reproductive toxicity, endocrine disruption, and an increased risk of cancer [41]. Recent studies indicate that, although DEP does not directly bind to estrogen receptor α (ERα), it can promote the proliferation of breast cancer cells via an ERα-mediated non-classical signaling pathway. Additionally, DEP may induce overexpression of cyclin D1, a key regulator of the G1 to S phase transition in the cell cycle [1]. Cyclin D1 overexpression is associated with rapid tumor growth, increased aggressiveness, and poor prognosis in breast cancer [42]. Research by Kononova and colleagues found that the concentration of DEP in the exhaled breath of CRC patients is significantly elevated, suggesting a close relationship between DEP and cancer metabolic processes [43]. However, the specific mechanisms by which DEP influences CRC remain unclear. This knowledge gap underscores the importance of our study, which seeks to elucidate how DEP contributes to CRC through network toxicology and molecular docking techniques. These approaches provide new insights for cancer prevention and treatment.

Using biological databases such as ChEMBL, OMIM, and TTD, we identified 225 target genes for DEP and 2,723 genes associated with CRC. By analyzing the intersection of these datasets, we identified 62 core genes as potential targets in DEP-induced CRC. Further analysis using the STRING database and Cytoscape software led to the construction of an interaction network, revealing five central targets: EP300, EZH2, HDAC1, HDAC2, and KDM1A. These genes are recognized as key targets in the DEP-induced CRC process.

EP300, a critical transcriptional coactivator, regulates chromatin accessibility through histone acetylation, enhancing the transcriptional activity of specific genes [44]. Recent studies have shown that EP300 promotes the expression of CSNK2A1 and accelerates CRC progression by activating the PI3K-AKT-mTOR axis [45]. Additionally, Zhou et al. demonstrated that EP300 acetylates MTA2 through its histone acetyltransferase activity, enhancing MTA2’s function and stability within cells. This modification promotes the growth and invasiveness of CRC cells [46]. Our GO and KEGG analyses further support EP300’s involvement in CRC, showing significant enrichment in the “Transcriptional Misregulation in Cancer” and “ATP-dependent Chromatin Remodeling” pathways. The enrichment of these pathways highlights EP300’s central role in regulating CRC, suggesting that DEP may facilitate CRC progression by upregulating EP300’s acetylation activity, altering chromatin structure, and abnormally activating oncogenes.

EZH2 is a core component of the Polycomb Repressive Complex 2 (PRC2), which primarily silences gene expression through the trimethylation of histone H3, leading to heterochromatin formation [47]. High expression of EZH2 is closely associated with poor prognosis in various cancers [48,49]. Studies have shown that the synergistic action of PRMT5 and EZH2 in CRC increases methylation at the promoter region of the CDKN2B gene. This epigenetic silencing leads to the downregulation of the tumor suppressor gene CDKN2B, thereby promoting cancer cell proliferation and invasiveness [50]. Additionally, Pappas et al. found that in breast cancer cells, the combined action of NOTCH and EZH2 suppresses the expression of the PTEN gene by enhancing H3K27me3 modification at its promoter region. This mechanism may contribute to the progression and aggressiveness of breast cancer [51]. In this study, GO enrichment analysis indicates significant involvement of EZH2 in epigenetic gene silencing, while KEGG analysis further suggests that EZH2 plays a crucial role in the “Polycomb Repressive Complex” and “Transcriptional Misregulation in Cancer” pathways. These findings imply that DEP may enhance EZH2 activity, leading to the suppression of tumor suppressor genes and increasing CRC cell invasiveness and survival capabilities.

HDAC1 and HDAC2 are key members of the histone deacetylase family, primarily inhibiting gene transcription by removing acetyl groups from histones, resulting in chromatin condensation [52]. These enzymes play a crucial role in CRC development and progression, with studies showing that high expression of HDAC2 is associated with increased proliferation and invasiveness in CRC. By suppressing the expression of the tumor suppressor gene TP53, HDAC2 enhances cancer cell drug resistance, promotes proliferation, and increases the potential for invasion and metastasis, thereby making the cancer more aggressive and harder to treat [53]. Furthermore, Chen et al. demonstrated that HDAC1 suppresses tumor suppressor gene expression in CRC through deacetylation, which enhances the stability and activity of HIF1α. This in turn activates the HIF1α/VEGFA signaling pathway, promoting tumor angiogenesis and cancer progression [54]. The findings in this study are consistent with existing literature regarding the roles of HDAC1 and HDAC2 in CRC. GO enrichment analysis reveals that both HDAC1 and HDAC2 are involved in the negative regulation of gene expression, inhibiting transcription at the epigenetic level through histone deacetylation, primarily as part of the histone deacetylase complex. In terms of molecular function, their deacetylase activity suppresses gene transcription, thus promoting cancer cell proliferation. KEGG analysis further highlights the critical role of HDAC1 and HDAC2 in the “Transcriptional Misregulation in Cancer” and “ATP-dependent Chromatin Remodeling” pathways. Their central role in chromatin structure regulation suggests that DEP may influence gene expression in CRC cells, particularly the expression of tumor suppressor genes, by upregulating or activating HDAC1 and HDAC2. This mechanism may reduce the expression of cell cycle inhibitors, allowing cancer cells to evade growth control.

KDM1A is a histone demethylase that regulates chromatin configuration and gene expression by removing methyl groups from histones, playing a crucial role in transcriptional regulation [55]. In CRC, high expression of KDM1A is closely associated with enhanced proliferation and invasiveness of cancer cells [56]. Studies have shown that the methylation level of the KDM1A promoter in CRC tissues is significantly lower than in normal tissues, and this hypomethylated state can increase the transcriptional activity of the KDM1A gene, potentially promoting tumor initiation and progression [57]. Additionally, Ding et al. found that high expression of KDM1A in CRC is strongly correlated with higher TNM stages and distant metastasis. Through epigenetic mechanisms, KDM1A downregulates the expression of the CDH1 gene, thereby promoting CRC proliferation and metastasis [58]. Our GO enrichment analysis indicates that KDM1A plays an essential role in regulating gene expression and chromatin configuration. As part of various transcriptional regulatory complexes, its histone demethylase activity is critical for the precise regulation of gene expression. KDM1A controls gene activity by modulating chromatin openness, a function that is particularly critical for the proliferation and metastasis of cancer cells. KEGG analysis further reveals that KDM1A is closely linked to the “chromatin remodeling” and “gene transcription regulation” pathways. The findings of this study not only support the significant role of KDM1A in chromatin structure and gene expression regulation but also emphasize its central role in CRC progression. These results suggest that DEP may alter gene expression by modulating KDM1A activity, thereby promoting the malignant characteristics of CRC.

Molecular docking is an advanced computational technique that simulates interactions between small molecules and larger biomolecules such as proteins and nucleic acids, predicting their binding modes and stability [59]. Similar to the study by He et al., which used network toxicology and molecular docking techniques to explore the interactions of common plasticizers such as DEP, Dimethyl phthalate (DMP), and Dioctyl phthalate (DOP) with breast cancer-related proteins, their findings showed that these chemicals could specifically bind to key proteins such as MAPK1, AKT1, SRC, ESR1, and ALB, potentially affecting the signaling pathways of breast cancer. Building on this approach, our study reveals that DEP binds with high affinity to key epigenetic regulatory proteins related to CRC, such as EP300, EZH2, HDAC1, HDAC2, and KDM1A [60]. This binding may not only directly affect the enzymatic activity and functional regulation of these proteins but also influence CRC development by regulating the expression of cancer-related genes.

This study suggests that DEP, a widely used plasticizer, may potentially influence CRC progression through interactions with key epigenetic regulators, including EP300, EZH2, HDAC1/2, and KDM1A, as predicted by network toxicology, molecular docking, and MD analyses. These computational results generate hypotheses that DEP exposure could modulate chromatin remodeling and transcriptional regulation, which are processes closely associated with CRC pathogenesis. However, the present study is entirely based on in-silico analyses, without experimental validation or positive-control benchmarking. The docking affinities observed (−6.6 to −5.7 kcal·mol ⁻ ¹) represent moderate binding strengths typical of small environmental molecules, and no direct biochemical or cellular evidence currently supports these predicted interactions. Future research should focus on validating these computational predictions through in vitro and in vivo experiments, as well as incorporating benchmarking approaches similar to those described by ULLah et al. [61] to contextualize binding affinities and refine mechanistic interpretation. Despite these limitations, this work provides a hypothesis-generating framework for understanding the potential molecular basis of DEP-associated carcinogenicity, offering valuable guidance for future studies on environmental exposure risk assessment, public health policy, and therapeutic development.

## Supporting information

S1 Fig Docking binding energy heat map of DEP with five core target proteins (EP300, EZH2, HDAC1, HDAC2, KDM1A). (JPG)

S2 Fig MD simulation results of the KDM1A–DEP complex. (A) RMSD of the complex, protein, and ligand; (B) Rg of the complex; (C) RMSF of protein residues; (D) distance between the binding site and ligand; (E) buried SASA; (F) superimposed conformations during simulation; (G) binding energy components (VDW and ELE); (H) per-residue binding energy contribution; and (I) variation in the number of hydrogen bonds during simulation.
(TIF)

S3 Fig MD simulation analysis of the EZH2–DEP complex. (A) RMSD of the complex, protein, and ligand; (B) Rg of the complex; (C) RMSF of protein residues; (D) distance between the binding site and ligand; (E) buried SASA; (F) superimposed conformations during simulation; (G) binding energy components (VDW and ELE); (H) per-residue binding energy contribution; and (I) variation in the number of hydrogen bonds during simulation.
(TIF)

S4 Fig MD simulation analysis of the EP300–DEP complex. (A) RMSD of the complex, protein, and ligand; (B) Rg of the complex; (C) RMSF of protein residues; (D) distance between the binding site and ligand; (E) buried SASA; (F) superimposed conformations during simulation; (G) binding energy components (VDW and ELE); (H) per-residue binding energy contribution; and (I) variation in the number of hydrogen bonds during simulation.
(TIF)

S5 Fig MD simulation analysis of the HDAC2–DEP complex. (A) RMSD of the complex, protein, and ligand; (B) Rg of the complex; (C) RMSF of protein residues; (D) distance between the binding site and ligand; (E) buried SASA; (F) superimposed conformations during simulation; (G) binding energy components (VDW and ELE); (H) per-residue binding energy contribution; and (I) variation in the number of hydrogen bonds during simulation.
(TIF)

S1 Table PPI network between DEP- and CRC-related target genes obtained from the STRING database, showing interacting protein pairs and combined scores. (XLSX)

S2 Table Topological parameters of shared DEP–CRC target genes in the PPI network, including degree, betweenness, closeness, and clustering coefficients used to identify core hub genes. (XLSX)

S3 Table Structural information of DEP target proteins used for molecular docking, including PDB identifiers, chain IDs, resolutions, experimental methods, and source organisms obtained from the RCSB PDB database. (XLSX)

S4 Table Summary of all databases and software used in this study, including names, versions, access dates, and source URLs to ensure methodological transparency and reproducibility. (XLSX)

S5 Table Predicted DEP-associated target genes obtained from ChEMBL, STITCH, and SwissTargetPrediction databases. (XLSX)

S6 Table CRC–associated genes collected from GeneCards, OMIM, and the TTD. (XLSX)

S7 Table List of 62 overlapping genes identified between DEP-associated targets and CRC–related genes. (XLSX)

S8 Table GO and KEGG enrichment analyses for the 62 overlapping genes between DEP-associated and CRC-related targets. (XLSX)

S9 Table Summary of molecular docking results between DEP and five key epigenetic regulators (HDAC1, KDM1A, EZH2, EP300, and HDAC2). (XLSX)

S1 File Detailed ADMET prediction results generated by ADMETlab, including physicochemical properties, absorption, distribution, metabolism, excretion, and general toxicity parameters. (PDF)

S2 File ProTox-based toxicity prediction results, including predicted LD_50_ values, toxicity classes, and endpoint-specific toxicological risks. (PDF)

## References

[1] Fiocchetti M, Bastari G, Cipolletti M, Leone S, Acconcia F, Marino M. The peculiar estrogenicity of diethyl phthalate: modulation of estrogen receptor α activities in the proliferation of breast cancer cells. Toxics. 2021;9(10):237. doi: 10.3390/toxics9100237 34678933 PMC8538674

[2] Mondal S, Basu S, Ghosh S, Guria S, Mukherjee S. Diethyl phthalate, a plasticizer, induces adipocyte inflammation and apoptosis in mice after long-term dietary administration. J Biochem Mol Toxicol. 2024;38(1):e23561. doi: 10.1002/jbt.23561 37942807

[3] González-Mariño I, Ares L, Montes R, Rodil R, Cela R, López-García E, et al. Assessing population exposure to phthalate plasticizers in thirteen Spanish cities through the analysis of wastewater. J Hazard Mater. 2021;401:123272. doi: 10.1016/j.jhazmat.2020.123272 32645544

[4] Katsikantami I, Tzatzarakis MN, Karzi V, Stavroulaki A, Xezonaki P, Vakonaki E, et al. Biomonitoring of bisphenols A and S and phthalate metabolites in hair from pregnant women in Crete. Sci Total Environ. 2020;712:135651. doi: 10.1016/j.scitotenv.2019.135651 31810691

[5] Li K, Liszka M, Zhou C, Brehm E, Flaws JA, Nowak RA. Prenatal exposure to a phthalate mixture leads to multigenerational and transgenerational effects on uterine morphology and function in mice. Reprod Toxicol. 2020;93:178–90. doi: 10.1016/j.reprotox.2020.02.012 32126281

[6] Xiong Z, Zeng Y, Zhou J, Shu R, Xie X, Fu Z. Exposure to dibutyl phthalate impairs lipid metabolism and causes inflammation via disturbing microbiota-related gut-liver axis. Acta Biochim Biophys Sin (Shanghai). 2020;52(12):1382–93. doi: 10.1093/abbs/gmaa128 33167028

[7] Aydemir D, Aydogan-Ahbab M, Barlas N, Ulusu NN. Effects of the in-utero dicyclohexyl phthalate and di-n-hexyl phthalate administration on the oxidative stress-induced histopathological changes in the rat liver tissue correlated with serum biochemistry and hematological parameters. Front Endocrinol (Lausanne). 2023;14:1128202. doi: 10.3389/fendo.2023.1128202 37274322 PMC10235726

[8] Ochieng J, Nangami GN, Ogunkua O, Miousse IR, Koturbash I, Odero-Marah V, et al. The impact of low-dose carcinogens and environmental disruptors on tissue invasion and metastasis. Carcinogenesis. 2015;36 Suppl 1(Suppl 1):S128-59. doi: 10.1093/carcin/bgv034 26106135 PMC4565611

[9] Desaulniers D, Vasseur P, Jacobs A, Aguila MC, Ertych N, Jacobs MN. Integration of epigenetic mechanisms into non-genotoxic carcinogenicity hazard assessment: focus on DNA methylation and histone modifications. Int J Mol Sci. 2021;22(20):10969. doi: 10.3390/ijms222010969 34681626 PMC8535778

[10] Duan M, Wang Y, Chen S, Lu J, Dong R, Yu Q, et al. Combined BPA and DIBP exposure induced intestinal mucosal barrier impairment through the notch pathway and gut microbiota dysbiosis in mice. Foods. 2025;14(2):214. doi: 10.3390/foods14020214 39856883 PMC11765467

[11] Pesonen M, Vähäkangas K. Contribution of common plastic-related endocrine disruptors to epithelial-mesenchymal transition (EMT) and tumor progression. Chemosphere. 2022;309(Pt 1):136560. doi: 10.1016/j.chemosphere.2022.136560 36152835

[12] Maiani E, Milletti G, Nazio F, Holdgaard SG, Bartkova J, Rizza S, et al. AMBRA1 regulates cyclin D to guard S-phase entry and genomic integrity. Nature. 2021;592(7856):799–803. doi: 10.1038/s41586-021-03422-5 33854232 PMC8864551

[13] Choi J, Jia G, Wen W, Shu X-O, Zheng W. Healthy lifestyles, genetic modifiers, and colorectal cancer risk: a prospective cohort study in the UK Biobank. Am J Clin Nutr. 2021;113(4):810–20. doi: 10.1093/ajcn/nqaa404 33675346 PMC8023827

[14] Murphy N, Moreno V, Hughes DJ, Vodicka L, Vodicka P, Aglago EK, et al. Lifestyle and dietary environmental factors in colorectal cancer susceptibility. Mol Aspects Med. 2019;69:2–9. doi: 10.1016/j.mam.2019.06.005 31233770

[15] Song M, Chan AT. Environmental factors, gut microbiota, and colorectal cancer prevention. Clin Gastroenterol Hepatol. 2019;17(2):275–89. 30031175 10.1016/j.cgh.2018.07.012 PMC6314893

[16] Sweeney C, Lazennec G, Vogel CFA. Environmental exposure and the role of AhR in the tumor microenvironment of breast cancer. Front Pharmacol. 2022;13:1095289. doi: 10.3389/fphar.2022.1095289 36588678 PMC9797527

[17] Hur SJ, Yoon Y, Jo C, Jeong JY, Lee KT. Effect of dietary red meat on colorectal cancer risk-a review. Compr Rev Food Sci Food Saf. 2019;18(6):1812–24. doi: 10.1111/1541-4337.12501 33336951

[18] Gautier L, Taboureau O, Audouze K. The effect of network biology on drug toxicology. Expert Opin Drug Metab Toxicol. 2013;9(11):1409–18. doi: 10.1517/17425255.2013.820704 23937336

[19] Wathieu H, Ojo A, Dakshanamurthy S. Prediction of chemical multi-target profiles and adverse outcomes with systems toxicology. Curr Med Chem. 2017;24(16):1705–20. doi: 10.2174/0929867323666161214115540 27978797

[20] Lee H, Kim J, Kim J-W, Lee Y. Recent advances in AI-based toxicity prediction for drug discovery. Front Chem. 2025;13:1632046. doi: 10.3389/fchem.2025.1632046 40698059 PMC12279745

[21] Zhang J, Li H, Zhang Y, Huang J, Ren L, Zhang C, et al. Computational toxicology in drug discovery: applications of artificial intelligence in ADMET and toxicity prediction. Brief Bioinform. 2025;26(5):bbaf533. doi: 10.1093/bib/bbaf533 41052279 PMC12499773

[22] Gaulton A, Hersey A, Nowotka M, Bento AP, Chambers J, Mendez D, et al. The ChEMBL database in 2017. Nucleic Acids Res. 2017;45(D1):D945–54. doi: 10.1093/nar/gkw1074 27899562 PMC5210557

[23] Szklarczyk D, Gable AL, Nastou KC, Lyon D, Kirsch R, Pyysalo S, et al. The STRING database in 2021: customizable protein-protein networks, and functional characterization of user-uploaded gene/measurement sets. Nucleic Acids Res. 2021;49(D1):D605–12. doi: 10.1093/nar/gkaa1074 33237311 PMC7779004

[24] Daina A, Michielin O, Zoete V. SwissTargetPrediction: updated data and new features for efficient prediction of protein targets of small molecules. Nucleic Acids Res. 2019;47(W1):W357–64. doi: 10.1093/nar/gkz382 31106366 PMC6602486

[25] Stelzer G, Rosen N, Plaschkes I, Zimmerman S, Twik M, Fishilevich S, et al. The GeneCards suite: from gene data mining to disease genome sequence analyses. Curr Protoc Bioinformatics. 2016;54:1.30.1-1.30.33. doi: 10.1002/cpbi.5 27322403

[26] Amberger JS, Bocchini CA, Scott AF, Hamosh A. OMIM.org: leveraging knowledge across phenotype-gene relationships. Nucleic Acids Res. 2019;47(D1):D1038–43. doi: 10.1093/nar/gky1151 30445645 PMC6323937

[27] Wang Y, Zhang S, Li F, Zhou Y, Zhang Y, Wang Z, et al. Therapeutic target database 2020: enriched resource for facilitating research and early development of targeted therapeutics. Nucleic Acids Res. 2020;48(D1):D1031–41. doi: 10.1093/nar/gkz981 31691823 PMC7145558

[28] Shannon P, Markiel A, Ozier O, Baliga NS, Wang JT, Ramage D, et al. Cytoscape: a software environment for integrated models of biomolecular interaction networks. Genome Res. 2003;13(11):2498–504. doi: 10.1101/gr.1239303 14597658 PMC403769

[29] Kanehisa M, Furumichi M, Sato Y, Kawashima M, Ishiguro-Watanabe M. KEGG for taxonomy-based analysis of pathways and genomes. Nucleic Acids Res. 2023;51(D1):D587–92. doi: 10.1093/nar/gkac963 36300620 PMC9825424

[30] Kanehisa M, Furumichi M, Sato Y, Matsuura Y, Ishiguro-Watanabe M. KEGG: biological systems database as a model of the real world. Nucleic Acids Res. 2025;53(D1):D672–7. doi: 10.1093/nar/gkae909 39417505 PMC11701520

[31] Gene Ontology Consortium. The Gene Ontology resource: enriching a GOld mine. Nucleic Acids Res. 2021;49(D1):D325–34. doi: 10.1093/nar/gkaa1113 33290552 PMC7779012

[32] Ashtiani M, Salehzadeh-Yazdi A, Razaghi-Moghadam Z, Hennig H, Wolkenhauer O, Mirzaie M, et al. A systematic survey of centrality measures for protein-protein interaction networks. BMC Syst Biol. 2018;12(1):80. doi: 10.1186/s12918-018-0598-2 30064421 PMC6069823

[33] Ugurlu SY, McDonald D, Lei H, Jones AM, Li S, Tong HY, et al. Cobdock: an accurate and practical machine learning-based consensus blind docking method. J Cheminform. 2024;16(1):5. doi: 10.1186/s13321-023-00793-x 38212855 PMC10785400

[34] Chai X, Jiang Y, Lu H, Huang X. Integrating ensemble machine learning and multi-omics approaches to identify Dp44mT as a novel anti-Candida albicans agent targeting cellular iron homeostasis. Front Pharmacol. 2025;16:1574990. doi: 10.3389/fphar.2025.1574990 40342996 PMC12058677

[35] Abraham MJ, Murtola T, Schulz R, Páll S, Smith JC, Hess B, et al. GROMACS: high performance molecular simulations through multi-level parallelism from laptops to supercomputers. SoftwareX. 2015;1–2:19–25. doi: 10.1016/j.softx.2015.06.001

[36] Maier JA, Martinez C, Kasavajhala K, Wickstrom L, Hauser KE, Simmerling C. ff14SB: Improving the accuracy of protein side chain and backbone parameters from ff99SB. J Chem Theory Comput. 2015;11(8):3696–713. doi: 10.1021/acs.jctc.5b00255 26574453 PMC4821407

[37] Marcisz M, Samsonov SA. Solvent model benchmark for molecular dynamics of glycosaminoglycans. J Chem Inf Model. 2023;63(7):2147–57. doi: 10.1021/acs.jcim.2c01472 36989082 PMC10091405

[38] Sousa da Silva AW, Vranken WF. ACPYPE - Antechamber python parser interface. BMC Res Notes. 2012;5:367. doi: 10.1186/1756-0500-5-367 22824207 PMC3461484

[39] Martín-Pozo L, Gómez-Regalado MDC, Moscoso-Ruiz I, Zafra-Gómez A. Analytical methods for the determination of endocrine disrupting chemicals in cosmetics and personal care products: a review. Talanta. 2021;234:122642. doi: 10.1016/j.talanta.2021.122642 34364451

[40] Jeong S-H, Jang J-H, Cho H-Y, Lee Y-B. Risk assessment for humans using physiologically based pharmacokinetic model of diethyl phthalate and its major metabolite, monoethyl phthalate. Arch Toxicol. 2020;94(7):2377–400. doi: 10.1007/s00204-020-02748-9 32303804

[41] Chiang C, Flaws JA. Subchronic exposure to di(2-ethylhexyl) phthalate and diisononyl phthalate during adulthood has immediate and long-term reproductive consequences in female mice. Toxicol Sci. 2019;168(2):620–31. doi: 10.1093/toxsci/kfz013 30649530 PMC6432868

[42] Chung J, Noh H, Park KH, Choi E, Han A. Longer survival in patients with breast cancer with cyclin d1 over-expression after tumor recurrence: longer, but occupied with disease. J Breast Cancer. 2014;17(1):47–53. doi: 10.4048/jbc.2014.17.1.47 24744797 PMC3988342

[43] Kononova E, Mežmale L, Poļaka I, Veliks V, Anarkulova L, Vilkoite I, et al. Breath fingerprint of colorectal cancer patients based on the gas chromatography-mass spectrometry analysis. Int J Mol Sci. 2024;25(3):1632. doi: 10.3390/ijms25031632 38338911 PMC10855950

[44] Ma L, Gao Z, Wu J, Zhong B, Xie Y, Huang W, et al. Co-condensation between transcription factor and coactivator p300 modulates transcriptional bursting kinetics. Mol Cell. 2021;81(8):1682-1697.e7. doi: 10.1016/j.molcel.2021.01.031 33651988

[45] Liu J. P300 increases CSNK2A1 expression which accelerates colorectal cancer progression through activation of the PI3K-AKT-mTOR axis. Exp Cell Res. 2023;430(1):113694. doi: 10.1016/j.yexcr.2023.113694 37391010

[46] Zhou J, Zhan S, Tan W, Cheng R, Gong H, Zhu Q. P300 binds to and acetylates MTA2 to promote colorectal cancer cells growth. Biochem Biophys Res Commun. 2014;444(3):387–90. doi: 10.1016/j.bbrc.2014.01.062 24468085

[47] Wasenang W, Puapairoj A, Settasatian C, Proungvitaya S, Limpaiboon T. Overexpression of polycomb repressive complex 2 key components EZH2/SUZ12/EED as an unfavorable prognostic marker in cholangiocarcinoma. Pathol Res Pract. 2019;215(7):152451. doi: 10.1016/j.prp.2019.152451 31126817

[48] Fan K, Zhang C-L, Qi Y-F, Dai X, Birling Y, Tan Z-F, et al. Prognostic value of EZH2 in non-small-cell lung cancers: a meta-analysis and bioinformatics analysis. Biomed Res Int. 2020;2020:2380124. doi: 10.1155/2020/2380124 33299862 PMC7705440

[49] Eichenauer T, Simmendinger L, Fraune C, Mandelkow T, Blessin NC, Kluth M, et al. High level of EZH2 expression is linked to high density of CD8-positive T-lymphocytes and an aggressive phenotype in renal cell carcinoma. World J Urol. 2021;39(2):481–90. doi: 10.1007/s00345-020-03200-4 32303902 PMC7910252

[50] Yang L, Ma D-W, Cao Y-P, Li D-Z, Zhou X, Feng J-F, et al. PRMT5 functionally associates with EZH2 to promote colorectal cancer progression through epigenetically repressing CDKN2B expression. Theranostics. 2021;11(8):3742–59. doi: 10.7150/thno.53023 33664859 PMC7914347

[51] Pappas K, Martin TC, Wolfe AL, Nguyen CB, Su T, Jin J, et al. NOTCH and EZH2 collaborate to repress PTEN expression in breast cancer. Commun Biol. 2021;4(1):312. doi: 10.1038/s42003-021-01825-8 33750924 PMC7943788

[52] Liu B, Chen S, Rose AL, Chen D, Cao F, Zwinderman M, et al. Inhibition of histone deacetylase 1 (HDAC1) and HDAC2 enhances CRISPR/Cas9 genome editing. Nucleic Acids Res. 2020;48(2):517–32. doi: 10.1093/nar/gkz1136 31799598 PMC6954403

[53] Alzoubi S, Brody L, Rahman S, Mahul-Mellier A-L, Mercado N, Ito K, et al. Synergy between histone deacetylase inhibitors and DNA-damaging agents is mediated by histone deacetylase 2 in colorectal cancer. Oncotarget. 2016;7(28):44505–21. doi: 10.18632/oncotarget.9887 27283986 PMC5190114

[54] Chen C, Wei M, Wang C, Sun D, Liu P, Zhong X, et al. The histone deacetylase HDAC1 activates HIF1α/VEGFA signal pathway in colorectal cancer. Gene. 2020;754:144851. doi: 10.1016/j.gene.2020.144851 32525044

[55] Nagasaka M, Tsuzuki K, Ozeki Y, Tokugawa M, Ohoka N, Inoue Y, et al. Lysine-specific demethylase 1 (LSD1/KDM1A) is a novel target gene of c-Myc. Biol Pharm Bull. 2019;42(3):481–8. doi: 10.1248/bpb.b18-00892 30828079

[56] Ding X, Zhang J, Feng Z, Tang Q, Zhou X. MiR-137-3p inhibits colorectal cancer cell migration by regulating a K DM1A-dependent epithelial-mesenchymal transition. Dig Dis Sci. 2021;66(7):2272–82. 32749639 10.1007/s10620-020-06518-6

[57] Zhong J, Pan R, Ying X, Wu B, Zhou C, Wu D, et al. Significant association between KDM1A promoter hypomethylation and colorectal cancer in Han Chinese. Pathol Res Pract. 2019;215(3):532–8. doi: 10.1016/j.prp.2018.12.005 30638951

[58] Ding J, Zhang Z-M, Xia Y, Liao G-Q, Pan Y, Liu S, et al. LSD1-mediated epigenetic modification contributes to proliferation and metastasis of colon cancer. Br J Cancer. 2013;109(4):994–1003. doi: 10.1038/bjc.2013.364 23900215 PMC3749561

[59] Naqvi AAT, Mohammad T, Hasan GM, Hassan MI. Advancements in docking and molecular dynamics simulations towards ligand-receptor interactions and structure-function relationships. Curr Top Med Chem. 2018;18(20):1755–68. doi: 10.2174/1568026618666181025114157 30360721

[60] He N, Zhang J, Liu M, Yin L. Elucidating the mechanism of plasticizers inducing breast cancer through network toxicology and molecular docking analysis. Ecotoxicol Environ Saf. 2024;284:116866. doi: 10.1016/j.ecoenv.2024.116866 39178760

[61] Ullah W, Uddin G, Rauf A, Khan MU, Akram Z, Shabbir CA, et al. Anti-inflammatory potential of Grewialin from Grewia optiva: insights from molecular docking, ADMET, DFT, and in-vitro studies. J Comput Aided Mol Des. 2025;39(1):56. doi: 10.1007/s10822-025-00632-1 40699260

