# Supplementary figures and images for: Elucidating key targets and mechanisms of diethyl phthalate-induced colorectal cancer through network toxicology and molecular docking
Source: PLoS One. 2026 Feb 17;21(2):e0343038. doi: 10.1371/journal.pone.0343038 (PMC12912549; doi:10.1371/journal.pone.0343038)

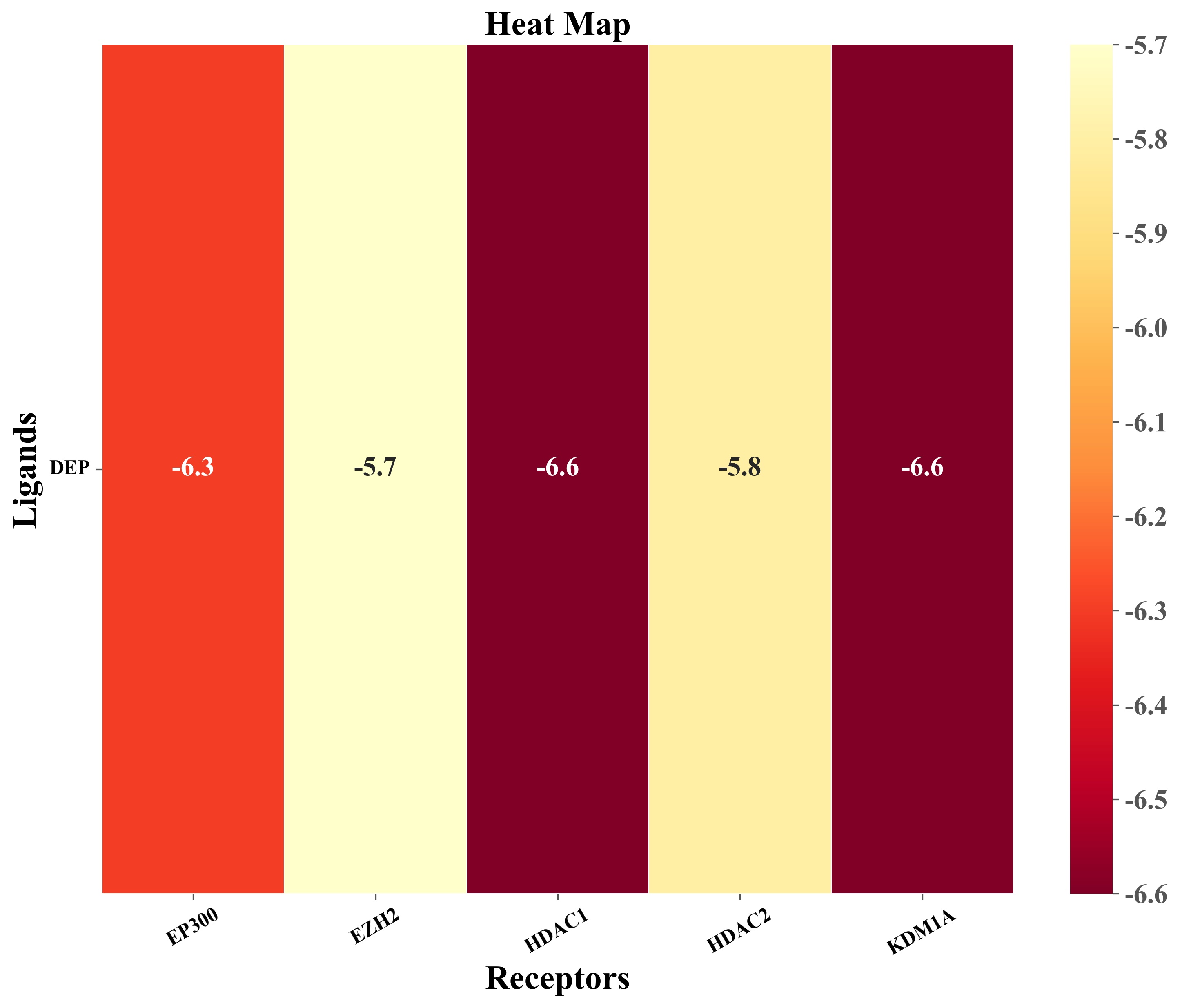

Supplement: S1 Fig — (JPG) [file pone.0343038.s001.jpg]

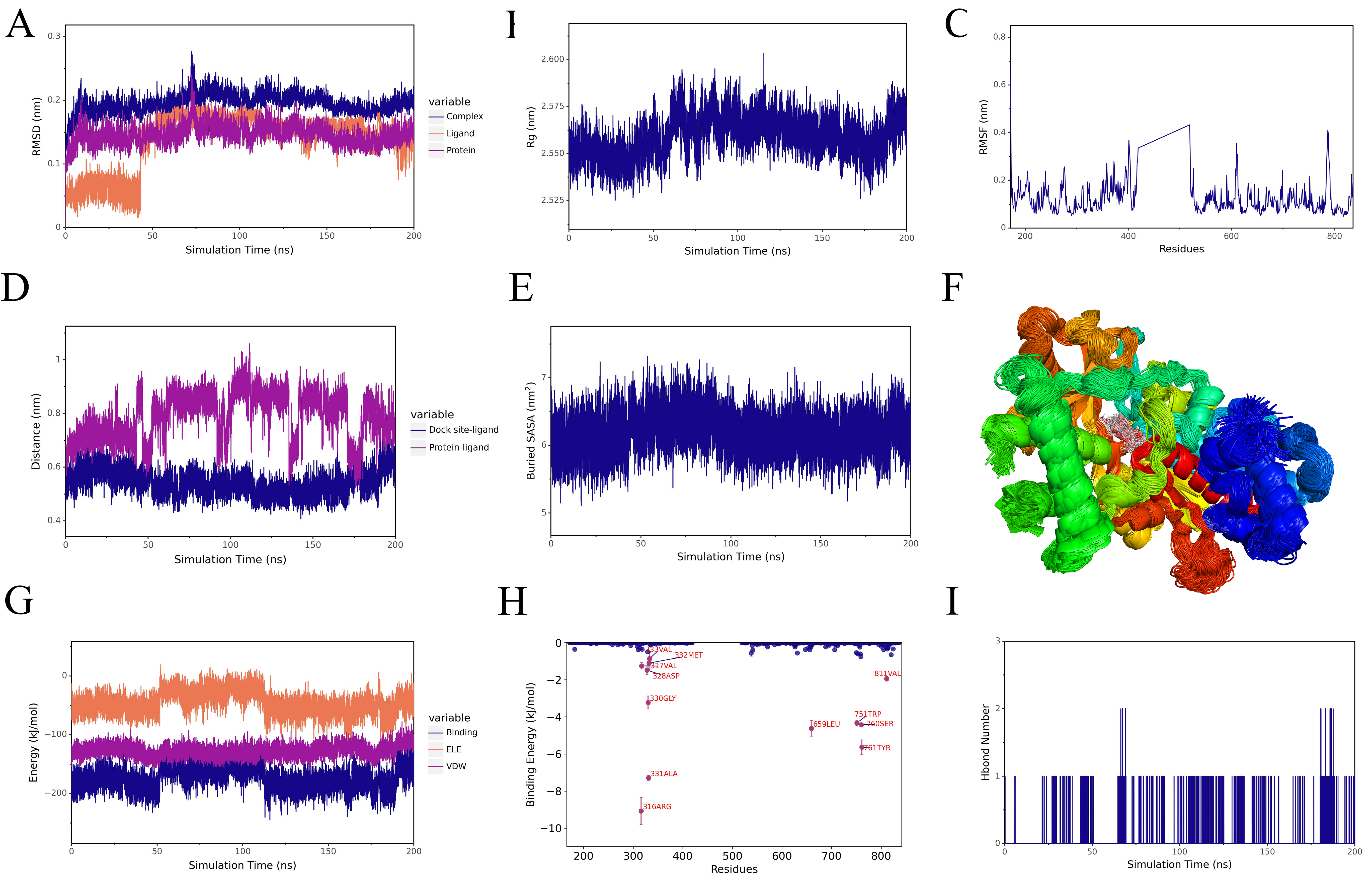

Supplement: S2 Fig — (A) RMSD of the complex, protein, and ligand; (B) Rg of the complex; (C) RMSF of protein residues; (D) distance between the binding site and ligand; (E) buried SASA; (F) superimposed conformations during simulation; (G) binding energy components (VDW and ELE); (H) per-residue binding energy contribution; and (I) variation in the number of hydrogen bonds during simulation. (TIF) [file pone.0343038.s002.tif]

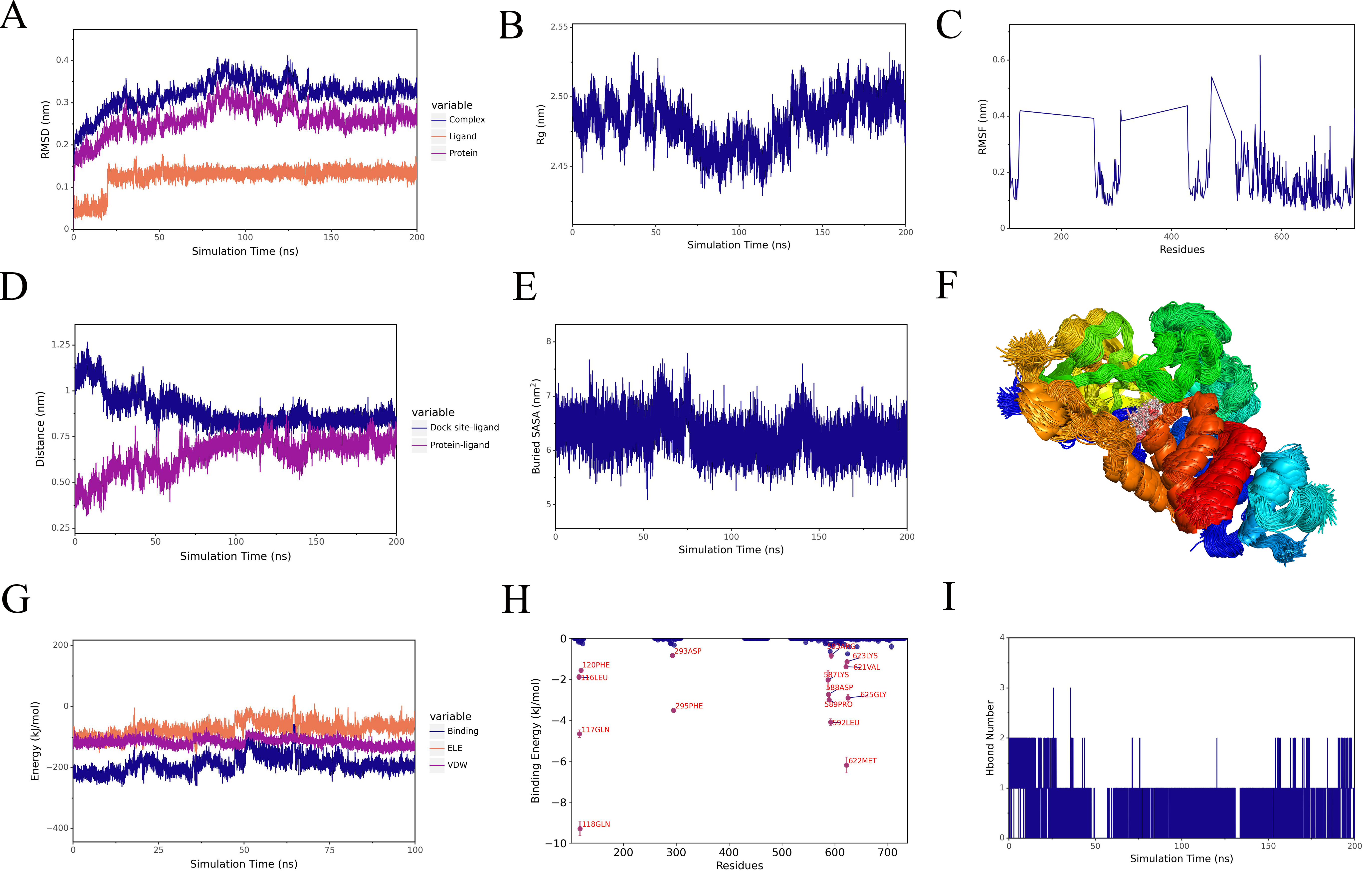

Supplement: S3 Fig — (A) RMSD of the complex, protein, and ligand; (B) Rg of the complex; (C) RMSF of protein residues; (D) distance between the binding site and ligand; (E) buried SASA; (F) superimposed conformations during simulation; (G) binding energy components (VDW and ELE); (H) per-residue binding energy contribution; and (I) variation in the number of hydrogen bonds during simulation. (TIF) [file pone.0343038.s003.tif]

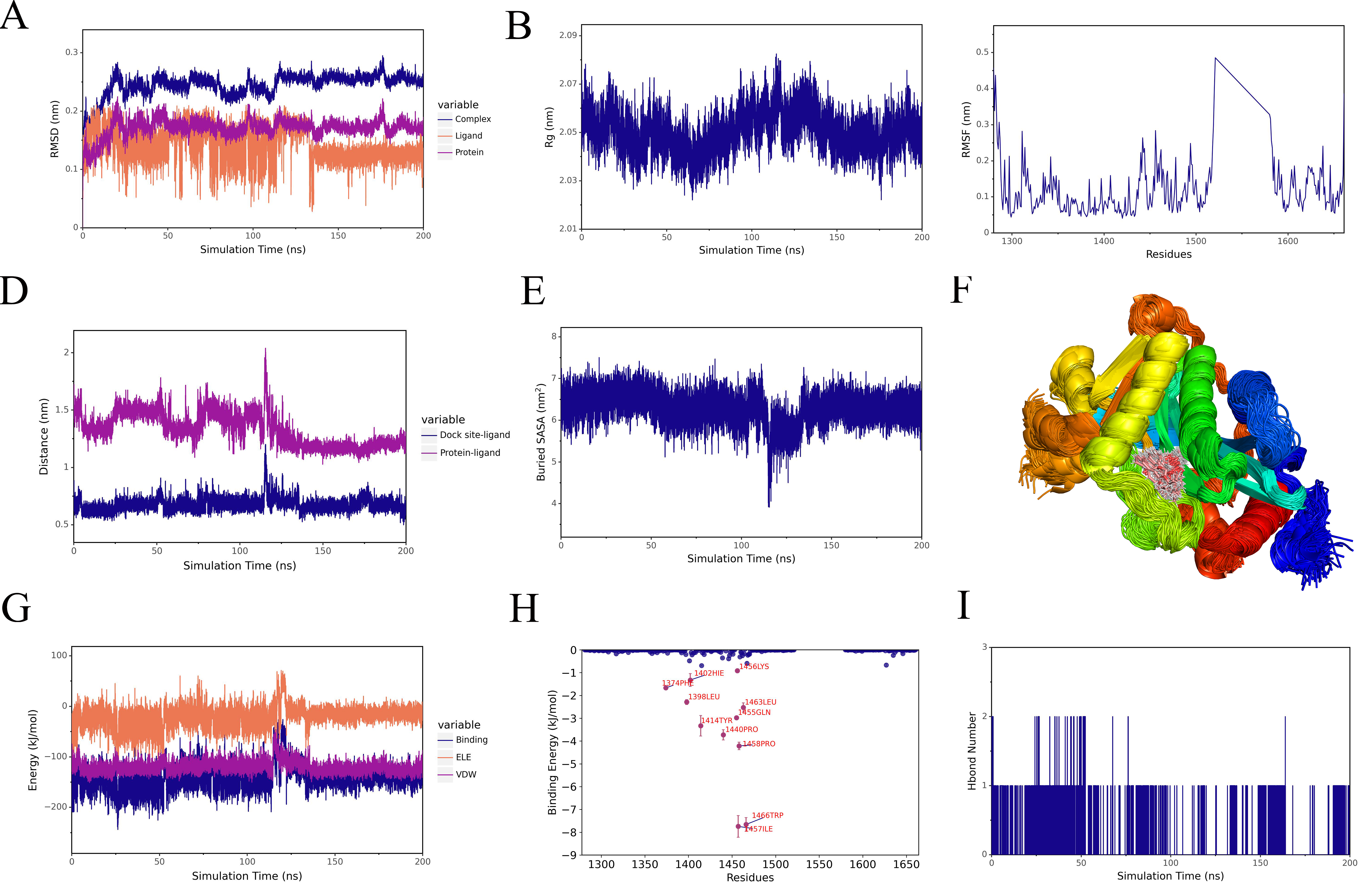

Supplement: S4 Fig — (A) RMSD of the complex, protein, and ligand; (B) Rg of the complex; (C) RMSF of protein residues; (D) distance between the binding site and ligand; (E) buried SASA; (F) superimposed conformations during simulation; (G) binding energy components (VDW and ELE); (H) per-residue binding energy contribution; and (I) variation in the number of hydrogen bonds during simulation. (TIF) [file pone.0343038.s004.tif]

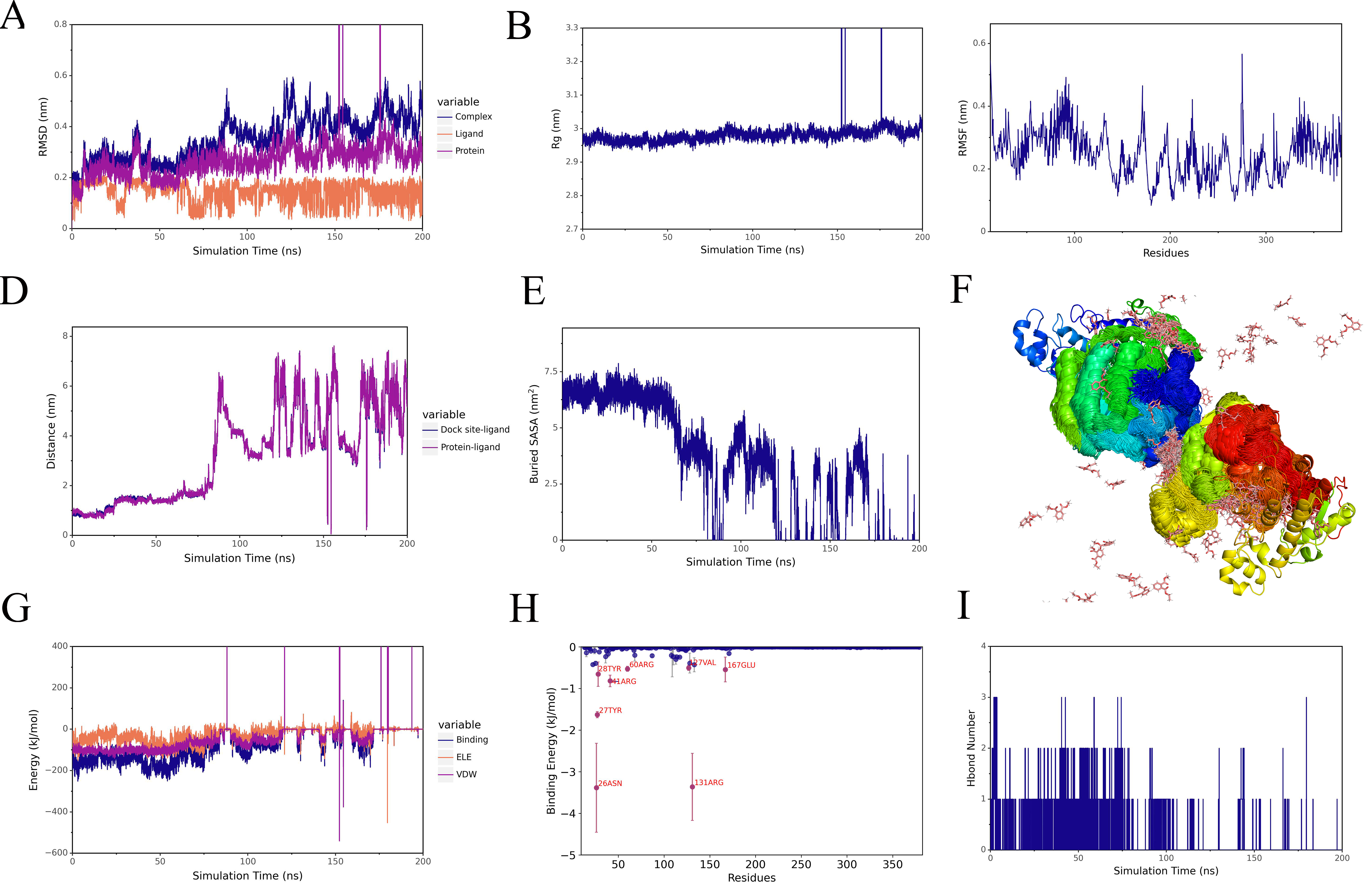

Supplement: S5 Fig — (A) RMSD of the complex, protein, and ligand; (B) Rg of the complex; (C) RMSF of protein residues; (D) distance between the binding site and ligand; (E) buried SASA; (F) superimposed conformations during simulation; (G) binding energy components (VDW and ELE); (H) per-residue binding energy contribution; and (I) variation in the number of hydrogen bonds during simulation. (TIF) [file pone.0343038.s005.tif]
